# THz Signal Generator Using a Single DFB Laser Diode and the Unbalanced Optical Fiber Interferometer

**DOI:** 10.3390/s20174862

**Published:** 2020-08-28

**Authors:** Blaž Pongrac, Denis Đonlagic, Matej Njegovec, Dušan Gleich

**Affiliations:** Faculty of Electrical Engineering and Computer Science, University of Maribor, Koroška Cesta 46, 2000 Maribor, Slovenia; denis.donlagic@um.si (D.Đ.); matej.njegovec@um.si (M.N.); dusan.gleich@um.si (D.G.)

**Keywords:** the optical signal frequency modulation, optical frequency sweep, optical fiber interferometer, THz band

## Abstract

This paper presents a frequency-modulated optical signal generator in the THz band. The proposed method is based on a fast optical frequency sweep of a single narrowband laser diode used together with an optical fiber interferometer. The optical frequency sweep using a single laser diode is achieved by generating short current pulses with a high amplitude, which are driving the laser diode. Theoretical analysis showed that the modulation frequency could be changed by the optical path difference of the interferometer or optical frequency sweep rate of a laser diode. The efficiency of the optical signal generator with Michelson and Fabry–Perot interferometers is theoretically analyzed and experimentally evaluated for three different scenarios. Interferometers with different optical path differences and a fixed optical frequency sweep rate were used in the first scenario. Different optical frequency sweep rates and fixed optical path differences of the interferometers were used in the second scenario. This paper presents a method for optical chirp generation using a programmable current pulse waveform, which drives a laser diode to achieve nonlinear optical sweep with a fixed optical path difference of the interferometer. The experimental results showed that the proposed signals could be generated within a microwave (1–30 GHz) and THz band (0.1–0.3 THz).

## 1. Introduction

In recent years, demand for high bandwidth, high frequency, and high-speed signal transmission has been increasing rapidly. The interest in the millimeter waves (mmW) and terahertz (THz) bandwidths has been increasing, in particular, the 0.1–10 THz frequency band [1], which has unique properties [2] that enable explosive detection, cancer screening, high-resolution imaging, and broadband communications.

Current techniques for THz generation and detection are expensive, due to lack of commercial transmitters and receivers, low loss THz waveguides, linear and broadband mixers, and high energy consumption and low efficiency of the components. Generating THz waves with optoelectrical methods is based mainly on the excitation of a photocurrent in crystals and semiconductors [3]. THz wave generators, based on photocurrent excitation, utilize Photoconductive Antennas (PCA), which can also be used for coherent THz wave detection for broadband signals and continuous wave signals, as the authors of [4] show in a theoretical model. The authors of [4] also show that, for coherent wave detection, the optical signal from the same source should illuminate both the PCA transmitter and PCA receiver. The amplitude and phase of THz waves can be estimated by changing the optical time delay between the PCA transmitter and PCA receiver, and measuring the DC component of the generated photocurrent in the PCA receiver. In current systems that utilize PCA for transmitting and receiving of THz waves, broadband optical signals are created using an fs-laser source, shown by the authors of [3], and continuous-wave optical signal with THz frequency is created by the beating of two continuous-wave optical signals with different wavelengths [1], generated by narrowband laser sources. Distributed Feedback (DFB) laser diodes with temperature control are utilized as narrowband laser sources. While fs-lasers are expensive and complex, the main drawback of temperature controlled DFB laser diodes is their low speed of wavelength tuning, as the whole laser diode is heated [5].

This paper proposes a new method for generating a frequency-modulated optical signal within the THz band that utilizes a fast Optical Frequency Sweep (OFS) of narrowband laser diode source and an unbalanced optical fiber interferometer. The proposed optical frequency modulation technique was analyzed analytically. The theoretical model showed that the modulation frequency depends on the Optical Path Difference (OPD) of the unbalanced optical fiber interferometer and OFS rate. The OPD of the unbalanced optical fiber interferometer can be controlled using a Piezoelectric (PZT) optical fiber stretcher [6]. However, the PZT fiber stretcher would be impractical for tuning the generated frequency with the proposed THz signal generator since the PZT fiber stretcher can stretch the fiber only for few millimeters with a fiber length of several meters [6]. The authors of [7] proposed a fast OFS of a standard telecommunication DFB laser diode with a selective and rapid temperature cycling of the DFB laser diode’s active region. The DFB laser diode’s active region was heated using a current pulse with a high amplitude and short duration. In [7,8,9], the authors proposed a linear wavelength sweep method based on the waveform modeling of a DFB laser diode’s driving current pulse, which can enable a modulation frequency tuning rate equal to the current pulse repetition rate. The main advantage of the proposed signal generator is the generation of complex waveforms, such as optical linear-frequency chirp signal using a special design of pulse current waveform, which drives a laser diode and produces a nonlinear OFS. Experimental results showed that signals up to 1.25 THz could be generated using the proposed design of a signal generator.

## 2. High-Frequency Optical Modulation

**Figure 1:**
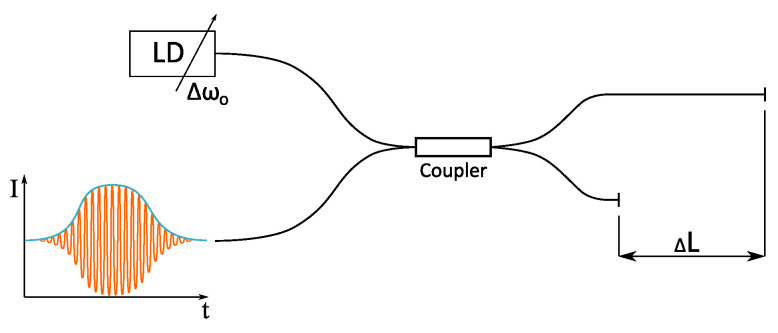
The proposed system utilizes an unbalanced fiber interferometer and Optical Frequency Sweep (OFS) narrowband laser diode.

The proposed optical signal generator, shown in Figure 1, consists of an unbalanced optical interferometer and a tunable laser source, which can perform rapid wavelength/optical frequency sweeps. The optical intensity $I_{r}$, back-reflected from the interferometer, can, in general, be described as

(1) $$I_{r}\left(t\right)=\frac{I_{0}}{2}\left(1+\cos\left(\frac{2n\Delta L\omega (t)}{c}\right)\right)$$

where $I_{0}$, *n*, ΔL, ω(t), and *c* represent incident intensity, an effective refractive index, the OPD of the interferometer, optical angular frequency, and speed of light, respectively. When an optical source with sweepable frequency is employed, the optical angular frequency becomes a function of time, and the phase in the cosine Function (1) is given by

(2) $$\phi \left(t\right)=\frac{2n\Delta L}{c}\left(\omega_{0}+\frac{\partial \omega }{\partial t}t\right)$$

where $\omega_{0}$ represents the initial angular frequency at the beginning of sweep, and ∂ω/∂t the optical angular frequency sweep rate. If the ∂ω/∂t is constant, i.e., ω(t) increases linearly over time, the power back-reflected from the interferometer will, thus, possess sinusoidal time characteristics. The temporal period *T* of the back-reflected signal can then be obtained from Equation (2) by setting the second term of Equation (2) to 2π and $\omega_{0}=0$:

(3) $$2\pi =\frac{2n\Delta L}{c}\left(\frac{\partial \omega }{\partial t}T\right)$$

An unbalanced interferometer, supplied with a linear frequency swept laser source, will thus generate a sinusoidal optical signal at its output, with a frequency *f* corresponding to

(4) $$f=\frac{2n\Delta L}{2\pi c}\frac{\partial \omega }{\partial t}=\frac{2}{c}n\Delta L\frac{\partial \nu }{\partial t}$$

where ∂ν/∂t represents OFS rate. The frequency at the output of an interferometer is set by a product of OFS rate ∂ν/∂t and the OPD ΔL of the optical interferometer. However, these two parameters are not arbitrary and must be selected according to the technical capabilities of the available devices. Optical Frequency Sweep Range (OFSR) and achievable OFS rate are always limited in practical laser sources. This implies that the duration $T_{D}$ of a linear frequency swept optical signal will always be limited in time in a realistic tunable laser, and is given by

(5) $$T_{D}=\frac{OFSR}{\frac{\partial \nu }{\partial t}}$$

This also implies that the length of generated frequency swept optical signals/waves will always be limited to $L_{D}=T_{D}\cdot c$. However, the limited length of optical swept signals limits the maximum allowable unbalancing (OPD ΔL) within the used interferometer, as the occurrence of the interference requires two optical signals to overlap in space. Therefore, the OPD ΔL must be smaller than the length of the generated swept optical signal $L_{D}$. Furthermore, as only overlapped signals generate an interference signal, we introduce an overlap efficiency factor *u*, which defines the fraction of the generated signal that is subjected to interface within the interferometer:

(6) $$u=\frac{L_{D}-n\Delta L}{L_{D}}=1-\frac{n\Delta L}{c\cdot OFSR}\frac{\partial \nu }{\partial t}$$

When *u* is close to 1, the entire generated swept wave/signal interferes within the interferometer, while at u=0 or negative values, the delay within the interferometer equals or exceeds the swept optical signal’s duration, which makes interference impossible. For example, if at least 50% overlap efficiency is desired, the Equation (6) shall be equal to 0.5 or

(7) $$\Delta L<c\frac{OFSR}{2n\frac{\partial \nu }{\partial t}}$$

By inserting the above condition into Equation (4), we obtained

(8) f=OFSR

The maximum frequency generated by the proposed principle is, thus, (theoretically) determined only by the sweep range of the tunable laser source. If Equation (8) is inserted back to Equation (4), the ΔL required to generate maximum frequency at the interferometer output is given by

(9) $$\Delta L=c\frac{OFSR}{2n\frac{\partial \nu }{\partial t}}=c\frac{T_{s}}{2n}$$

where $T_{s}$ is a duration of the frequency sweep given by

(10) $$T_{s}=\frac{OFSR}{\frac{\partial \nu }{\partial t}}$$

It should be stressed that, when slow-scanning rate tunable laser sources are used, considerable OPD might be required to achieve the frequency predicted by Equation (8), which might be difficult for realization due to the losses, polarization sensitivity, and potential mechanical (vibration) influences. Thus, the present principle is particularly suitable for generating high-frequency RF/THz signals when using rapidly tunable laser sources that can perform sweeps within a few microseconds, preferably in the nanosecond ranges, in order to keep the required interferometer unbalancing below the km range. Finally, it should be stressed that the above analysis is made for a linear frequency sweep laser source. When the optical frequency is changed nonlinearly over time, it might provide an opportunity for generation of modulated optical signal, thus forming more complex waveforms.

## 3. The OFS Signal Generator

**Figure 2:**
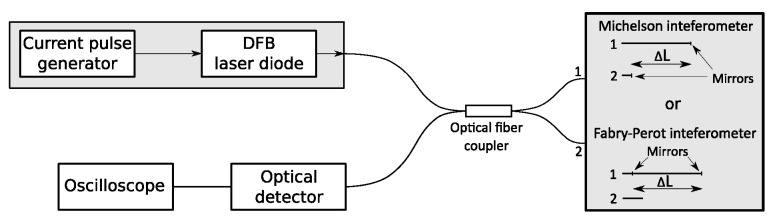
Set-up of proposed frequency modulator with Michelson fiber interferometer and Fabry–Perot fiber interferometer.

The proposed OFS signal generator is shown in Figure 2. It consists of an unbalanced fiber interferometer (Michelson or Fabry–Perot interferometer), which allows a compact and robust device realization, a single DFB laser diode, and a current pulse generator utilized for a fast OFS of the DFB laser diode. Based on Equation (8), the theoretical maximal generated frequency *f* with the proposed signal generator is limited to OFSR=1.25 THz at overlap efficiency factor u=0.5. Optical Frequency modulated signals were detected using the Keysight N7004A optical detector and UXR0334A oscilloscope.

### 3.1. Unbalanced Interferometer Design

#### 3.1.1. Optical Interferometer Design

The proposed OFS signal generator uses a Michelson or Fabry–Perot unbalanced optical fiber interferometer, as shown in Figure 2. In-line semireflective mirrors in the Fabry–Perot interferometers were fabricated using RF sputtering of high refractive index material, such as TiO_2_, and were spliced together with a fusion splicer employing a suitable program [10]. The Michelson interferometers were fabricated using RF-sputtering of Al on an optical fiber coupler’s arms. The in-line semireflective mirrors used in the Fabry–Perot interferometers reached ~10% reflectivity, while the mirrors used in the Michelson interferometers reached ~30% reflectivity. The achieved mirror reflectivity depends on manufacturing process and material selection.

#### 3.1.2. Interferometer Parameters

A generated signal frequency *f* using optical frequency modulation principle Equation (4) depends on the OPD ΔL and OFS rate ∂ν/∂t. The Michelson and Fabry–Perot fiber interferometers were fabricated considering the limitation that frequency sweep duration $T_{s}$ was changed between 200 and 400 ns, and generated frequencies were in a range between 1 GHz and 300 GHz. Seven interferometers, different in length, were designed for the target frequencies, shown in Table 1, for a frequency sweep duration of $T_{s}=250$ ns. The same physical interferometers were used at a frequency sweep duration of 200 ns and 400 ns. Corresponding frequencies were recalculated using Equation (4) and reported in Table 2. The performances of all seven fabricated interferometers are evaluated in the experimental results section.

### 3.2. Current Pulse Generator

A particularly suitable and highly cost-efficient swept laser source, which can accommodate the requirements presented by theoretical modeling, was obtained using a standard Telecom DFB laser diode driven by unusually high-amplitude and short-duration current pulses. This method was studied in detail in [7]. The method utilizes selective and very rapid temperature cycling of the DFB laser diode’s active region. A custom-designed high-speed programmable current generator drove the DFB diode based on the work in [8,9], where the authors modeled the current pulse waveform its duration and peak amplitude used for current control of the DFB diode to achieve linear OFS. Temperature recuperation of the DFB laser diode’s active region limits the pulse repetition rate in the proposed current pulsed generator [7]. A pulse repetition rate of 1.5 kHz was used.

**Figure 3:**
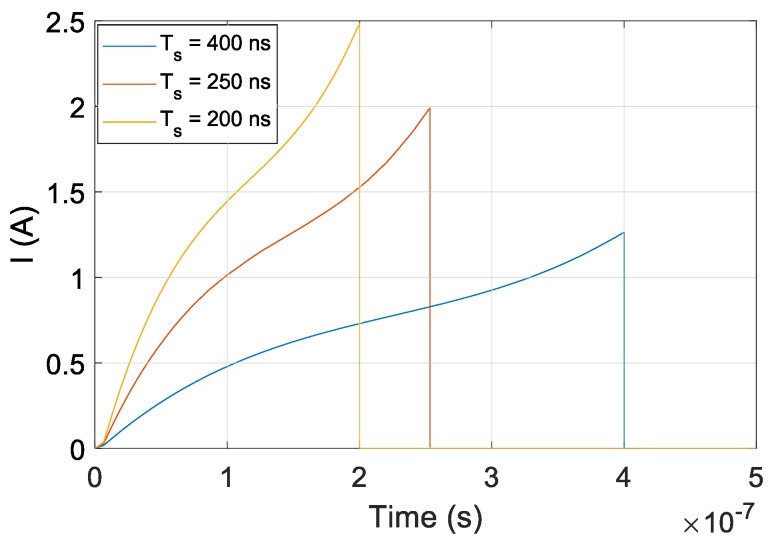
Current pulse waveforms for $T_{s}=200$ ns, $T_{s}=250$ ns and $T_{s}=400$ ns.

The optical frequency shift of the DFB laser diode is not proportional to temperature change in DFB laser diode’s active region [8]. The authors of [8] experimentally determined the current waveform to achieve linear OFS. The current waveform I(t), shown in Figure 3, can be modeled as a linear function $I_{\nu }\left(t\right)$ and nonlinear part $I_{DFB}\left(t\right)$, given by

(11) $$I\left(t\right)=I_{\nu }\left(t\right)+I_{DFB}\left(t\right)$$

where $I_{\nu }\left(t\right)$ represents OFS as a linear function, i.e., OFS rate ∂ν/∂t is constant function over time, and $I_{DFB}\left(t\right)$ represents the DFB laser diode’s characteristics. We assume that $I_{DFB}\left(t\right)$ has the same waveform for any type of current waveform. $I_{DFB}\left(t\right)$ was experimentally determined in [8]. Optical frequency shift modeling is difficult without knowing exact physical parameters of DFB laser diode. The waveform presented in Figure 3 was deramped (linear part was removed) and the nonlinear part of the current waveform $I_{DFB}$ for $T_{s}=250$ ns was approximated using 6th order polynomial given by

(12) $$I_{DFB}\left(a\right)=12.6811a^{6}-41.4872a^{5}+52.552a^{4}-28.8854a^{3}+3.922a^{2}+1.246a-0.0243$$

where variable *a* represents a time variable, normalized to a duration of a waveform $a=t/T_{s}$.

Different waveforms can be generated by changing a linear part of the Equation (11), as presented in Section 3.2.2.

#### 3.2.1. Current Waveform for Linear OFS

To generate a signal with a single frequency using the proposed optical signal generator, the OFS rate is constant over time. Our method is based on the assumption that the same amount of a charge is applied to the DFB laser diode at different $T_{s}$ to ensure a constant OFSR. This can be achieved when a product of $T_{s}\cdot I\left(T_{s}\right)/2$ has a constant value. If a peak current amplitude $I(T_{s})$ applied to the DFB diode was $I(T_{s})=2$ A at $T_{s}=250$ ns [9], then the peak amplitudes $I(T_{s})$ for sweep duration times $T_{s}$ of 200, 250, and 400 ns were set to 2.5, 2, and 1.25 A, respectively. Current pulse waveforms for providing a linear OFS using OFS duration times $T_{s}$ of 200, 250, and 400 ns are as presented in Figure 3.

#### 3.2.2. Current Waveform for Nonlinear OFS

**Figure 4:**
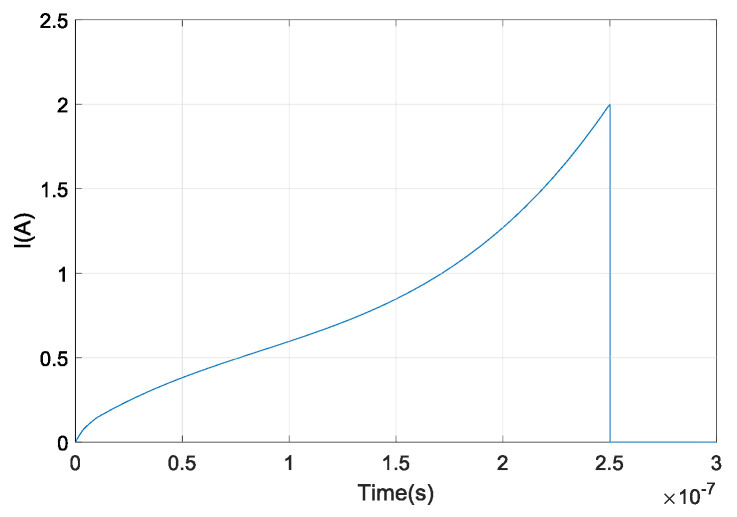
Current pulse waveform for generating a chirped signal with current pulse duration of $T_{s}=250$ ns.

The theoretical model for phase modulation in unbalanced interferometers Equation (2) shows that the OFS rate ∂ν/∂t is time-dependent. To generate a linear chirp waveform its frequency should increase linearly Equation (4). This can be achieved by modeling the OFS rate as a linear function. Different optical waveforms can be generated using the proposed OFS generator by current waveform modeling Equation (11). In this paper, we propose to model the OFS rate ∂ν/∂t as a linear function in the form of κ·t. To obtain a current waveform $I_{\nu }\left(t\right)$, the OFS rate should be integrated over a certain time:

(13) $$I_{\nu }\left(t\right)=\int_{0}^{t}\frac{\partial \nu }{\partial t}dt=\int_{0}^{t}\kappa tdt=\frac{1}{2}\cdot \kappa \cdot t^{2}$$

where κ is a normalized constant. The current waveform I(t) for generation of optical linear chirp signal is represented by a sum of quadratic function $I_{\nu }\left(t\right)$ and a DFB diode’s characteristic function $I_{DFB}\left(t\right)$, shown in Figure 4.

### 3.3. Temperature Stability of the Optical Interferometers

The ambient temperature impacts on interferometer’s OPD, polarization, and dispersion in the single-mode optical fiber. The OPD can be modeled as

**Figure 5:**
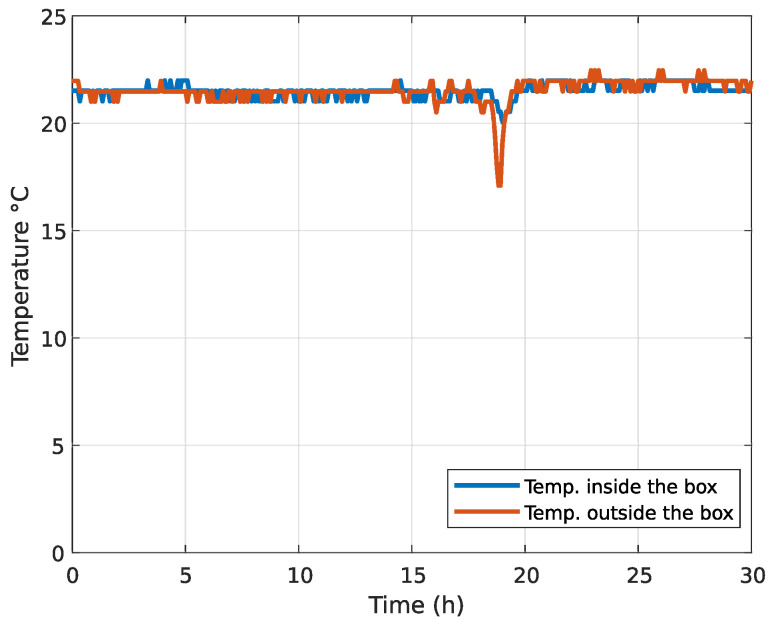
Temperature stability test: The temperature inside the isolated box and temperature outside the isolated box were measured every 5 min for 30 h.

(14) OPD=nΔL+ΔOPD

where ΔOPD is the temperature depended term given by

(15) $$\Delta OPD=\Delta L\frac{dn}{dT}\Delta T$$

where ΔT is the temperature change and dn/dT is the thermo-optical coefficient. It should be stressed that a typical value of dn/dT for silica-based optical fibers is in order of $10^{-5}$. With the change of temperature for 10 K, changes to the ΔOPD are in the order of $10^{-4}$ at OPD=1 m. Mathematical model Equation (4) shows that in optical fiber interferometer with longer OPDs there is no significant impact on the generated frequency by the change of temperature. The polarization and dispersion effects are very difficult to model. In the literature there has not been extensive research made on environmental impacts on optical fiber interferometers with ultra-long OPD. Based on that, we made a compromise and placed the interferometers into the isolated box. A temperature stability test was performed on an isolated box. The temperature on the inside and outside of the box was measured every 5 min for 30 h. The results are presented in Figure 5. Two temperature shifts were introduced into the temperature stability test. The temperature inside the box held both temperature swings for 30 min. It was concluded that the isolating box has sufficient temperature stability in the laboratory environment; therefore, the impact of temperature swings on the optical fiber interferometer should be minimal.

## 4. Experimental Evaluation of the OFS Signal Generator

The generated signal’s frequency using the OFS signal generator is changed by an OPD ΔL or OFS rate ∂ν/∂t. The proposed OFS signal generator was evaluated using three scenarios, where the target frequency was changed and overlap efficiency was evaluated. In the first scenario, a fixed OFS rate ∂ν/∂t at $T_{s}=250$ ns and fiber interferometers with different OPD ΔL were used, as reported in Table 1. The second scenario considered a constant OPD ΔL=0.16 m and sweep duration times $T_{s}$ of 200, 250, and 400 ns, respectively. In the third scenario, the OFS rate ∂ν/∂t was modeled as a linear function of time and fixed OPD ΔL=0.02 m was used to generate linear-frequency optical chirp signal.

### 4.1. Constant $T_{s}$ at Different ΔL

**Figure 6:**
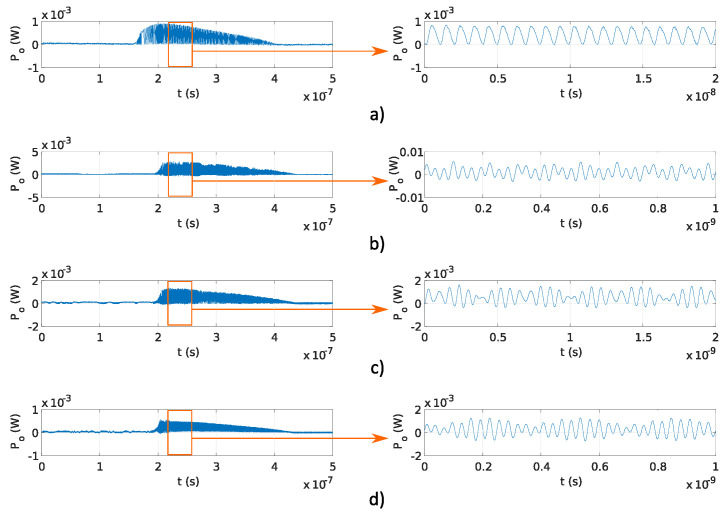
Pulses and fringes generated using Fabry–Perot interferometers with different OPD, reported in Table 1: (**a**) 1 GHz, (**b**) 8 GHz, (**c**) 15 GHz, and (**d**) 30 GHz.

**Figure 7:**
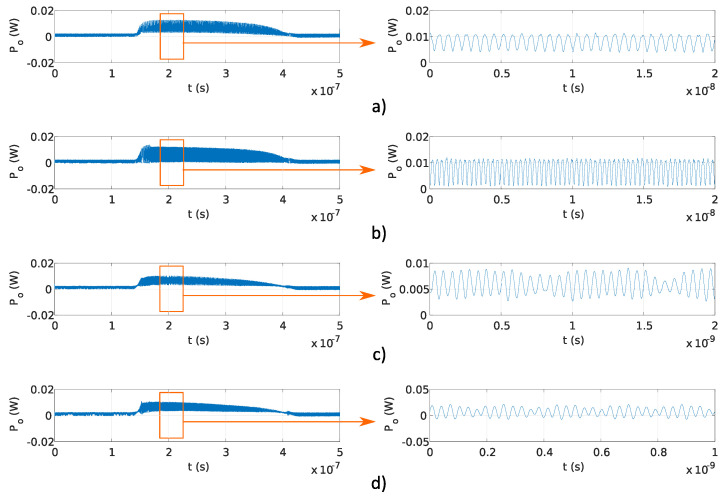
Pulses and fringes generated using Michelson interferometers with different OPD, reported in Table 1: (**a**) 1 GHz, (**b**) 8 GHz, (**c**) 15 GHz, and (**d**) 30 GHz.

In this experiment, a fixed OFS rate ∂ν/∂t and different OPD ΔL were used to generate the signals with target frequencies, as reported in Table 1. The stability of generated frequency and overlap efficiency of the generated optical signal were evaluated within the microwave and THz band. In the preliminary experiments, OPD ΔL were used, which correspond to f≤30 GHz, reported in Table 1. The results obtained using the Fabry–Perot interferometers and results obtained using the Michelson interferometers are shown in Figure 6a–d and Figure 7a–d, respectively. The pulse amplitude is not constant over time, as shown in Figure 6 and Figure 7. The reason for that is the drop of the emitted optical power from the DFB laser diode. The emitted optical power from the DFB laser diode increases at the start of the pulse, and the temperature in the DFB laser diode’s active region is increasing as well, causing lower photon emission in the DFB laser diode’s active region. Therefore, the emitted DFB laser diode’s optical power starts to decrease with the increase in temperature and reaches zero value at the end of the current pulse ($T_{s}$). The delay $t_{D}$ was lower than the sampling time of used equipment and could not be detected in Figure 6 and Figure 7. Frequency modulated optical signals obtained with Michelson interferometers have higher peak optical power $P_{o}$, as the mirrors used in Michelson interferometers have higher reflectivity, as described in Section 3.1.1. Amplitude differences in signals generated using Fabry–Perot interferometers and shown in Figure 6 are caused by the tolerances made in a manual design of optical fiber interferometer.

Measured frequencies $f_{M}$ and $f_{FP}$ for signals generated using Michelson and Fabry–Perot interferometers, reported in Table 3, are stable. The difference between target frequency *f* and measured frequencies $f_{M}$ and $f_{FP}$ can be attributed to differences in the practical implementation of the interferometer and reported OPD ΔL from Table 1. The accuracy of the measured frequencies depends on architecture and manufacturing process of the interferometers. The Fabry–Perot fiber interferometer consists of a single optical fiber with two in-line mirrors and is spliced to the optical fiber coupler. The Michelson interferometer consists of mirrors at the end of two optical fibers that are spliced to optical fiber coupler’s arms. While using the same procedure for optical fiber preparation and RF sputtering, higher accuracy of the OPD can be achieved at building the Fabry–Perot interferometer as the Fabry–Perot interferometer is built from a single optical fiber. However, with longer OPD ΔL, the difference in OPD’s accuracy becomes negligible with the available optical fiber interferometer manufacturing procedure.

**Figure 8:**
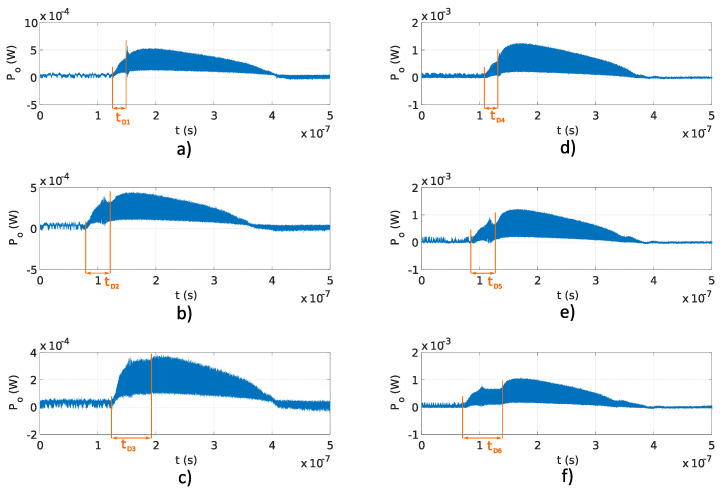
Pulses generated using Fabry–Perot and Michelson interferometer with OPD reported in Table 1: (**a**) Fabry–Perot interferometer, f=0.1 THz, $u_{1}=0.95$; (**b**) Fabry–Perot interferometer, f=0.2 THz, u=0.92; (**c**) Fabry–Perot interferometer, f=0.3 THz, u=0.88; (**d**) Michelson interferometer, f=0.1 THz, u=0.96; (**e**) Michelson interferometer, f=0.2 THz, u=0.91; (**f**) Michelson interferometer, f=0.3 THz, u=0.88.

Figure 8a–c and Figure 8d–f show signals generated using Fabry–Perot and Michelson interferometers using the ΔL reported in Table 1, which corresponds to frequencies *f* above 0.1 THz. The delay $t_{D}$ is more obvious in signals obtained using the Michelson interferometers than the Fabry–Perot interferometers. In the Michelson fiber interferometer, the optical pulse travels in two different interferometer arms, which resulted in two in amplitude equal reflected pulses the point of interference. While in the Fabry–Perot interferometer, optical pulses travel in a single optical fiber and reflected signals are not equal in amplitude at the point of the interference. Because of the difference in amplitude in reflected pulses in the Fabry–Perot interferometer, delay $t_{D}$ is visible in longer interferometers. Overlap efficiency factor *u* for each measurement shown in Figure 8a–f was estimated using the expression $u=1-t_{D}/\left(2\cdot T_{s}\right)$, which was derived using Equation (6), where $t_{D}/\left(2\cdot T_{s}\right)$ represents the fraction of the reflected pulse without interference. A comparison between analytical overlap efficiency factor *u* (6), and measured overlap efficiency factors $u_{FP}$ and $u_{M}$ are reported in Table 4 for signals generated using the Fabry–Perot and Michelson interferometers shown in Figure 8a–c and Figure 8d–f, respectively. The measured overlap efficiency factors $u_{FP}$ and $u_{M}$ are compliant with the analytical overlap efficiency factors *u*. Differences between measured overlap efficiency factors $u_{FP}$ and $u_{M}$ and estimated overlap efficiency factor *u* are due to deviations between the analytical and real OPD ΔL. The delay $t_{D}$ depends on the OPD ΔL, as shown in Equation (6).

### 4.2. Constant ΔL at Different $T_{s}$

**Figure 9:**
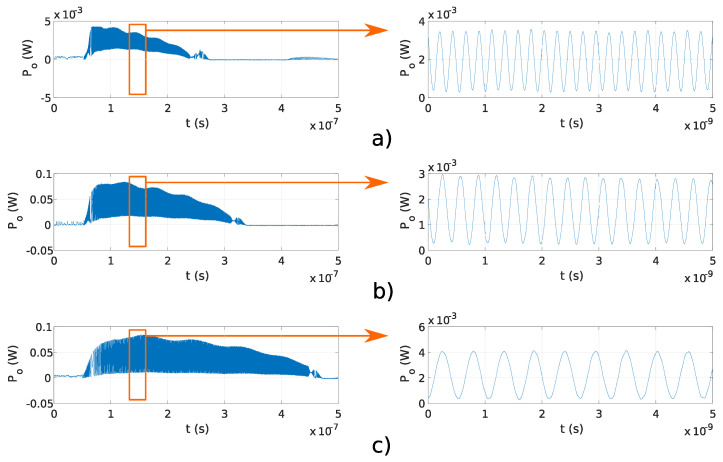
Pulses and fringes measured with the Michelson fiber interferometer using ΔL=0.16 m and (**a**) $T_{s}=200$ ns, (**b**) $T_{s}=250$ ns, (**c**) $T_{s}=400$ ns.

**Figure 10:**
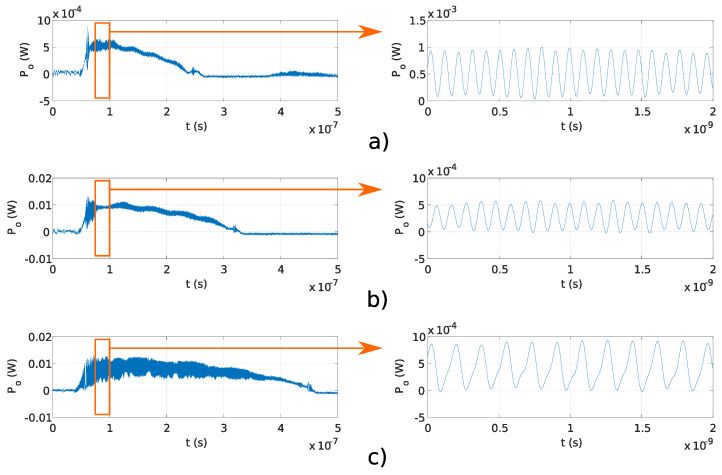
Pulses and fringes obtained using Fabry–Perot fiber interferometer with OPD ΔL=0.16 m and (**a**) $T_{s}=200$ ns, (**b**) $T_{s}=250$ ns, (**c**) $T_{s}=400$ ns.

The main advantage of the proposed system is the configurable OFS rate ∂ν/∂t, which is as fast as the current pulse repetition rate. Programmable frequency tuning of optical signal generator was evaluated experimentally using Michelson and Fabry–Perot interferometers with fixed OPD ΔL=0.16 m, and current pulses with durations $T_{s}$ of 200, 250, and 400 ns. Figure 9a–c and Figure 10a–c show the results obtained using Michelson fiber and Fabry–Perot fiber interferometers. Frequency modulation is visible on all measurements, and the delay $t_{D}$ is lower than the sampling time of used equipment; therefore, it was not detected.

Signal frequencies $f_{M}$ and $f_{FP}$ obtained using Michelson and Fabry–Perot interferometers at different values of $T_{s}$ for target frequency *f* determined using Equation (4) and ΔL=0.16 m are reported in Table 5. The difference between estimated frequencies *f* and measured frequencies $f_{M}$ and $f_{FP}$ due to differences in the actual OPDs from the estimated OPDs ΔL=0.16 m from Table 1.

A rise time $\tau_{R}$ of the optical pulse fed into the Fabry–Perot and Michelson interferometers is defined as a time difference between 10 % and 90 % of optical signal maximal value. Table 6 presents rising times $\tau_{R}$ of optical pulses for $T_{s}$ of 200, 250, and 400 ns, shown in Figure 9 and Figure 10. The rising times $\tau_{R}$ for optical pulses with time duration $T_{s}$ of 200, 250, and 400 ns, presented in Table 6, are increasing with current duration times. It can be concluded that the rising time of the optical pulse is shorter with shorter current pulse by maintaining the same energy of the DFB laser diode’s current pulse, as presented in Section 3.2.1.

### 4.3. Optical Linear Chirp Signal at Constant ΔL and Constant $T_{s}$

**Figure 11:**
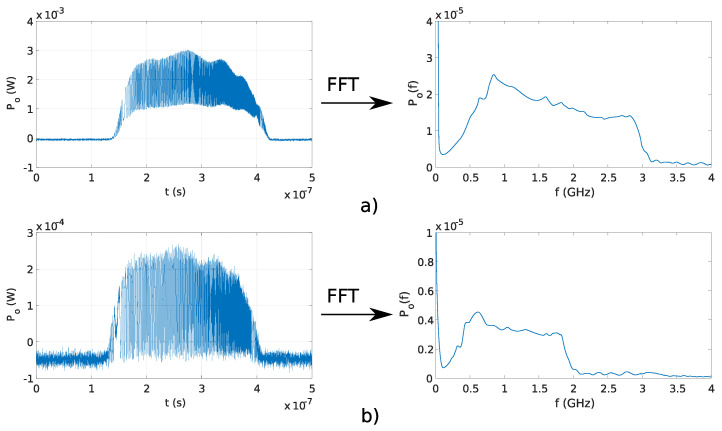
Generated chirp signals ant their spectrum: (**a**) with Michelson fiber interferometer where OPD ΔL=0.033 m and (**b**) Fabry–Perot fiber interferometer where OPD ΔL=0.02 m.

Complex waveforms can be generated using the proposed signal generator, if OFS rate ∂ν/∂t is a function of time. The current pulse waveform for generating linear chirp was designed and used with Michelson fiber interferometer with OPD ΔL=0.033 m and Fabry–Perot fiber interferometer with OPD ΔL=0.02 m. Figure 11a,b shows the generated signals with Michelson and Fabry–Perot interferometers, respectively. The maximal frequency in signal, generated using the Michelson interferometer, was 3 GHz, and maximal frequency of the signal generated with Fabry–Perot interferometer was 2 GHz. The amplitudes of generated optical linear chirp signal decreases over time. Optical power, emitted from DFB laser diode, starts to drop with the rise of the temperature. The amplitude is gradually falling with rising frequency since higher frequencies are generated toward the end of the generated optical pulse.

### 4.4. Hardware Limitations

The current implementation of the proposed signal generator is limited in sampling time/frequency of digital-to-analog converter and peak of the current waveform. The theoretical maximal generated signal’s frequency using the proposed system is 1.25 THz at overlap efficiency factor u=0.5. Implementation of 1.25 THz signal using the proposed system requires a $T_{s}$ of 50 ns and peak of the current waveform of 13 A at OPD of ΔL=5.08 m. The hardware in present form can generate pulses with a duration of 150 ns and a maximal current of 3.5 A, which at OPD of 15.25 m resulted in the generated signal’s frequency of 1.25 THz. However, with longer interferometers, polarization effects in optical fiber could impact the amplitude of generated optical signal.

The proposed method can be characterized as a broadband signal generator. The proposed generator’s linewidth and phase noise parameters depend on the generated optical pulse, which is generated using an appropriate driving current waveform. Linewidth and phase noise depend on the current pulse generator. Linewidth could be determined as the bandwidth of generated current waveform $B=1/T_{s}$, where $T_{s}$ is a current pulse duration, and phase noise of generated optical pulse depends on the current pulse generator’s phase noise. Repeatability and stability of generated optical signals with the proposed method rely on the clock stability of the current pulse generator and can be managed accordingly.

The Keysight UXR0334A oscilloscope, used for measuring the generated optical signals, has a 33 GHz bandwidth and uses downconversion, and the Keysight N7004A optical detector is limited to 33 GHz bandwidth and 8 mW input optical power. Because of the downconversion and lower amplitudes of optical signals, lower harmonics of the sampling frequency are present in the measured signal. Due to bandwidth limitations of measuring equipment, the proposed optical signal generator was evaluated within microwave band, where generated signal is visible and can be analyzed, and THz band, where generated signal’s envelope is visible.

## 5. Conclusions

This paper presented a new method for generating signals using modulation of optical signals within the THz band. The proposed modulator was designed using a single DFB laser diode and a fiber interferometer. The idea was to change the current through the DFB laser diode to obtain the OFS signal and use the fiber interferometer to modulate the optical signal with a frequency in the THz spectrum.

Theoretical analysis of the proposed method showed that the frequency of a generated signal could be changed (i) by optimizing the OPD ΔL and (ii) by changing the OFS rate. Bandwidth is limited by the difference in the optical path of a fiber interferometer and is determined by the overlap efficiency factor. If the overlap efficiency factor is 0.5, then the bandwidth is limited within the OFSR. The proposed method was evaluated experimentally using three scenarios. In the first scenario, the fixed OFS rate was chosen, and, in the second scenario, the OPD ΔL was chosen. With a fixed OFS rate and several different interferometers, frequency modulation and overlap efficiency were evaluated experimentally. At a chosen OPD ΔL, OFS was changed programmatically, and the generated signal was examined. Generated signals obtained using the first two scenarios were compliant with the theoretical analysis of the proposed system. However, choosing the fixed OPD ΔL is more practical. Fast changes of the OPD ΔL are impractical, while changes of OFS rate can be as fast as the pulse repetition rate of the proposed current pulse generator, and can be performed using the proposed experimental set-up. A single harmonic can be generated with a linear OFS, where the OFS rate is constant. Nevertheless, the theoretical model shows that a more complex waveform can be generated if the OFS rate is a function of time. In the third evaluation scenario, a linear chirp was generated at fixed OPD ΔL and a fixed current pulse duration $T_{s}$. Therefore, the proposed signal generator can enable fast and controllable chirping of optical signals in the THz band.

The proposed system design resulted in a smaller number of electronic components, simple architecture, and low-cost implementation of a frequency-modulated optical signal generator capable of generating a single harmonic and signals with complex waveforms within the THz band, which could be used with PCA for generating and detecting THz waves. In future work, a method for generating THz waves using the proposed optical signal generator and PCAs should be investigated.

## Figures and Tables

**Table 1:** Fabry–Perot and Michelson interferometers OPD ΔL for target frequency *f*.

*f*(GHz), $T_{s}=250$ ns	1	8	15	30	100	200	300
ΔL (m)	0.02	0.16	0.3	0.61	2.03	4.06	6.1

**Table 2:** Estimated frequencies for $T_{s}=200$ ns and $T_{s}=400$ ns and OPDs ΔL from Table 1.

ΔL (m)	0.02	0.16	0.3	0.61	2.03	4.06	6.1
*f* (GHz), $T_{s}=200$ ns	1.2	9.9	18.6	37.3	124.2	248.4	372.6
*f* (GHz), $T_{s}=400$ ns	0.6	5	9.3	18.6	62.1	124.2	186.3

**Table 3:** Measured frequencies $f_{M}$ and $f_{FP}$ obtained using Michelson and Fabry–Perot interferometers with OPDs reported in Table 1.

Target Frequency *f* (GHz)	$f_{M}$ (GHz)	$f_{\mathrm{FP}}$ (GHz)
1	1.6	1.02
8	6.25	7.9
15	16.3	15.3
30	29.2	31.1

**Table 4:** Theoretical overlap efficiency factor *u* estimated using Equation (6) and measured overlap efficiency factors $u_{FP}$ and $u_{M}$ from signals shown in Figure 8a–f, obtained using Fabry–Perot and Michelson interferometer, respectively.

Target Frequency	Overlap Efficiency Factors		
*f* (THz)	*u*	$u_{M}$	$u_{\mathrm{FP}}$
0.1	0.96	0.96	0.95
0.2	0.92	0.91	0.92
0.3	0.88	0.88	0.88

**Table 5:** Measured frequencies $f_{M}$ and $f_{FP}$ obtained using Michelson and Fabry–Perot interferometers with OPD ΔL=0.16 m and $T_{s}$ of 200, 250, and 400 ns.

$T_{s}$ (ns)	*f* (GHz)	$f_{M}$ (GHz)	$f_{\mathrm{FP}}$ (GHz)
200	9.9	8.8	10.3
250	8	6.3	7.9
400	5	3.7	5.6

**Table 6:** Measured rise times $\tau_{R}$ obtained with $T_{s}$ of 200, 250 and 400 ns.

$T_{s}$ (ns)	$\tau_{R}$ (ns)
200	8.83
250	14.2
400	17.6
